# Alcohol-Induced Liver Injury Is Modulated by Nlrp3 and Nlrc4 Inflammasomes in Mice

**DOI:** 10.1155/2013/751374

**Published:** 2013-12-16

**Authors:** David A. DeSantis, Chih-wei Ko, Yang Liu, Xiuli Liu, Amy G. Hise, Gabriel Nunez, Colleen M. Croniger

**Affiliations:** ^1^Departments of Nutrition, Case Western Reserve University School of Medicine, 10900 Euclid Avenue, Cleveland, OH 44106, USA; ^2^Department of Anatomic Pathology, Cleveland Clinic, Cleveland, OH 4406, USA; ^3^Department of Pathology, Case Western Reserve University School of Medicine, Cleveland, OH 44106, USA; ^4^Department of Medicine, Louis Stokes Cleveland Department of Veterans' Affairs Medical Center, Cleveland, OH 44106, USA; ^5^Center for Global Health and Diseases, Case Western Reserve University, Cleveland, OH 44106, USA; ^6^Department of Pathology, University of Michigan Medical School, Ann Arbor, MI 48109, USA

## Abstract

Alcoholic liver disease (ALD) is characterized by increased hepatic lipid accumulation (steatosis) and inflammation with increased expression of proinflammatory cytokines. Two of these cytokines, interleukin-1**β** (IL-1**β**) and IL-18, require activation of caspase-1 via members of the NOD-like receptor (NLR) family. These NLRs form an inflammasome that is activated by pathogens and signals released through local tissue injury or death. NLR family pyrin domain containing 3 (Nlrp3) and NLR family CARD domain containing protein 4 (Nlrc4) have been studied minimally for their role in the development of ALD. Using mice with gene targeted deletions for Nlrp3 (Nlrp3^−/−^) and Nlrc4 (Nlrc4^−/−^), we analyzed the response to chronic alcohol consumption. We found that Nlrp3^−/−^ mice have more severe liver injury with higher plasma alanine aminotransferase (ALT) levels, increased activation of IL-18, and reduced activation of IL-1B. In contrast, the Nlrc4^−/−^ mice had similar alcohol-induced liver injury compared to C57BL/6J (B6) mice but had greatly reduced activation of IL-1**β**. This suggests that Nlrp3 and Nlrc4 inflammasomes activate IL-1**β** and IL-18 via caspase-1 in a differential manner. We conclude that the Nlrp3 inflammasome is protective during alcohol-induced liver injury.

## 1. Introduction

Alcoholic liver disease (ALD) represents a variety of clinical and morphological changes that range from steatosis to inflammation and necrosis (alcoholic hepatitis) to progressive fibrosis (alcoholic cirrhosis) [1]. Most chronic heavy drinkers exhibit steatosis characterized by a greater amount of macrovesicular fat content than microvesicular fat. In addition, hepatocyte ballooning degeneration with mixed lobular inflammation is evident [2, 3]. Patients with ALD also have elevated serum concentrations of alanine aminotransferase (ALT) and aspartate aminotransferase (AST), which is evidence of liver injury. The severity of disease is not always correlated with the amount of alcohol consumed. In fact, most long-term heavy drinkers develop steatosis, but only 20–30% of these patients develop hepatitis, and less than 10% will progress to cirrhosis [4–6].

Activation of the immune system plays a critical role in the pathogenesis of ALD. Presently the current hypothesis for ethanol-induced liver injury proposes that ethanol results in leakage of bacterial products from the gut. Furthermore, chronic ethanol exposure alters the jejunal microflora leading to an increase in Gram-negative bacteria. Together these alterations cause an increase in circulating lipopolysaccharide (LPS) from Gram-negative bacteria in alcoholics [7].

The integrated human immune response has traditionally been divided into 2 branches: innate and adaptive (or acquired) immunity. The innate immune system is responsible for the initial task of recognizing and eradicating potentially dangerous microorganisms. A critical property of the innate immune system is its ability to discriminate microbes from itself through recognition of conserved microbial structures called “pathogen”-associated molecular patterns (PAMPs) such as LPS, peptidoglycan, flagellin, and microbial nucleic acids [8].

Recognition of PAMPs is accomplished by membrane bound Toll-like receptors (TLRs) and cytoplasmic nucleotide oligomerization domain-like receptors (NLRs) [9]. The mammalian NLR family is composed of >20 members that contain a C-terminal leucine-rich repeat domain, a central nucleotide-binding NACHT domain, and a N-terminal protein-protein interaction domain composed of a caspase activation and recruitment domain or pyrin domain [10]. These proteins promote the assembly of multiprotein complexes, termed inflammasomes, which are required for the activation of inflammatory caspases. Upon sensing of PAMPs, NLR forms a complex with the effector molecule, procaspase-1 with or without the contribution of an adapter molecule apoptosis-associated speck-like Card-domain containing protein (ASC) [11–13]. Assembly of the inflammasome complex leads to cleavage of procaspase-1 to its active form of caspase-1. Once activated, caspase-1 promotes proteolytic maturation and activation of IL-1*β*, IL-18, and caspase-7 as well as deactivation of IL-33 [9] to mediate pyroptosis or cell death [14].

Nlrp3 and Nlrc4 are the best characterized NLR molecules. Nlrp3 controls caspase-1 activation in response to a range of stimuli such as ATP, pore-forming toxins, or uric acid crystals [15–17]. Nlrc4 is important for the activation of caspase-1 in macrophages infected with several pathogenic bacteria including *Salmonella enteric* server Typhimurium (*Salmonella*), *Legionella pneumophila *(*Legionella*), and *Pseudomonas aeruginosa* (*Pseudomonas*) [18–23]. However the role of these NLR molecules in the development of ALD has been investigated minimally. Because activation of proinflammatory cytokines is increased in ALD [24], we hypothesized that deletion of the inflammasome would prevent development of ALD. In this paper we analyzed the role of Nlrp3 and Nlrc4 in the development of ALD using the Lieber-DeCarli ethanol-containing diet model in B6, Nlrp3^−/−^, and Nlrc4^−/−^ mice.

## 2. Experimental Procedures

### 2.1. Husbandry

Nlrp3^−/−^ (generous gift from Dr. Amy G. Hise) and Nlrc4^−/−^ (generous gift from Dr. Gabriel Nunez) mice were maintained at Case Western Reserve University. All mice were in the C57BL/6J (B6) background. The control B6 mice were originally from Jackson labs but have been bred and maintained at Case Western Reserve University for over 8 generations. Mice were raised in microisolator cages with a 12 hour light: 12 hour dark cycle. All mice were weaned at 3-4 weeks of age and raised on LabDiet number 5010 autoclavable rodent chow (LabDiet, Richmond, IN) *ad libitum* until studies were initiated.

### 2.2. Ethanol Feeding Diet Study and Ethanol Gavage

Eight to ten week-old female B6, Nlrp3^−/−^, and Nlrc4^−/−^ mice were fed either Lieber-DeCarli ethanol-containing diet (EtOH-Fed) or pair-fed control diet (Pair-Fed) (Dyets Inc., Bethlehem, PA). Mice were randomized into ethanol-fed and pair-fed groups and then adapted to control liquid diet for 2 days. The ethanol-fed group was allowed free access to ethanol-containing diet with increasing concentrations of ethanol: 1% (vol/vol) and 2% for 2 days, then 4% ethanol for 7 d, and finally 5% ethanol for a further 2 weeks. For chronic alcohol study, we measured the volume of ethanol-containing diet consumed daily and fed the control mice pair-fed diets which isocalorically substituted maltose dextrin for ethanol over the entire feeding period. For measurements of serum ethanol concentrations, blood was taken from the tail vein 2 hours into the feeding cycle. At the end of the feeding trial, mice were sacrificed and blood was collected by cardiac puncture. Plasma was isolated using Microtainer plasma separator tubes (Becton Dickinson, Franklin Lakes, NJ). For acute administration of ethanol, rates of ethanol clearance were determined using a spectrophotometric enzyme assay [25]. Female mice were administered an oral gavage of ethanol (5 g ethanol/kg body weight of ethanol) as described in [25, 26]. Blood samples (50 uL) were taken from tail vein (at 30 min post injection) and serum was isolated. The serum was added to 2 mL 3% perchloric acid and centrifuged for 10 min at 1000 ×*g*. Resulting supernatants were used to determine serum ethanol concentration using an alcohol dehydrogenase enzyme assay as described in [25]. Female mice were used for this study because they are more susceptible to alcohol-induced liver injury and have a significantly higher risk of developing cirrhosis for any given level of alcohol intake [27].

### 2.3. Hepatic Triglycerides, Plasma Alanine Aminotransferase (ALT) Activity

For measurement of liver triglycerides, we saponified 100–200 mg of liver with an equal volume by weight of 3 M KOH/65% ethanol as described by Salomon and Flatt [28]. We measured glycerol concentration against glycerol standards using a commercially available triglyceride glycerol phosphate oxidase (GPO) reagent set (Pointe Scientific, Lincoln Park, MI) as previously described in [29]. We measured alanine aminotransferase (ALT) using commercially available enzymatic assay kit (Sigma-Aldrich, St. Louis, MO) as per manufacturer's directions.

### 2.4. Liver Histology and Inflammatory Score

Formalin-fixed tissues were paraffin-embedded, sectioned, coded, and stained with hematoxylin and eosin. Histological examinations were performed in a blinded fashion by our experienced pathologist (Xiuli Liu, M.D., Anatomic Pathology, Cleveland Clinic, Cleveland, OH) with histological scoring system for NAFLD [30]. Steatosis and inflammation scores ranged from 0 to 3 with 0 being within normal limits and 3 being the most severe.

### 2.5. Real-Time Quantitative Reverse Transcription PCR (Real-Time qRT-PCR)

Total RNA from 30 mg of liver was isolated with an RNeasy Mini kit (Qiagen, Valencia, CA) and synthesized to single-strand cDNA from 500 ng of total RNA using random hexamer primers and MMTV reverse transcriptase (Applied Biosystems, Foster City, CA). Real-time qRT-PCR analysis was performed using Bullseye EvaGreen SYBR qPCR reagent (MidSci, St. Louis, MO) on a Chromo4 Cycler (MJ Research/Bio-Rad, Hercules, CA) using specific primer sequences (see Supplementary Table 1 in supplementary in supplementary material available online at http://dx.doi.org/10.1155/2013/751374). Data was normalized using the comparative Ct method with load variations normalized to 18S rRNA. A ΔΔCT  value is obtained by subtracting control ΔCT values from experimental ΔCT. The ΔΔCT values are converted to fold difference compared to control by raising two to the ΔΔCT power [31–33].

### 2.6. TNF-*α*, IL-1*β*, and IL-18 ELISA

The concentration of TNF-*α* in the liver was assessed using an enzyme-linked immunosorbent assay (ELISA) binding assay from liver protein homogenate derived from pair-fed and ethanol treated mice at the end of the feeding study as previously described [34]. The TNF-*α* ELISA was performed according to manufacturer's directions (BioLegend, San Diego, CA). The concentration of TNF-*α* was normalized by liver weight used for protein homogenization. Plasma isolated from pair-fed and ethanol-fed animals was used to measure IL-1*β* (R&D Systems, Minneapolis, MN) and IL-18 (Affymetrix eBioscience, San Diego, CA) by ELISA according to manufacturer's directions.

### 2.7. Protein Isolation and Western Blotting

Proteins were isolated and western blot analysis was performed from liver samples as previously described [34]. The membranes were incubated with antibodies to CYP2E1 (1 : 10,000; Fitzgerald Industries International Concord, MA), P-STAT (1 : 5,000; Abcam, Cambridge, MA), and total STAT3 (1 : 5,000; Cell Signaling Technology, Danvers, MA). The immunoreactive proteins were detected using the SuperSignal West Pico Chemiluminescent Substrate Kit (Thermo Scientific, Rockford, IL) and the density of the immunoreactive bands was measured by scanning densitometry (UN-SCAN-IT gel software, Orem, Utah). The membranes were stripped using ReView Buffer Solution (Amresco, Solon, OH) and normalized for loading differences using heat shock cognate-70 (HSC70) (1 : 16,000; Santa Cruz Biotechnology, Santa Cruz, CA) as previously described [34].

### 2.8. Caspase-3/7 Activity

Caspase-3/7 activity in the liver was assessed using an ELISA binding assay from protein homogenate derived from ethanol-fed mice livers at the completion of the feeding study. The assay was performed according to manufacturer's directions (Promega, Madison, WI) and was normalized by homogenate protein concentration.

### 2.9. Hydroxyproline Assay

Hydroxyproline content in liver was measured as previously described [35]. Briefly, liver tissues were homogenized in phosphate buffered saline and then hydrolyzed for 4 hours in 0.5 mL of 12 N Hydrochloric acid at 120°C. A portion of the hydrolysate (5 ul) was mixed with citrate/acetate buffer (238 mM citric acid, 1.2% glacial acetic acid, 532 mM sodium acetate, and 85 mM sodium hydroxide). Chloramine-T reagent (100 uL) was added (0.282 g chloramine-T into 16 mL sodium/acetate buffer, 2 mL n-Propanol, and 2 mL ddH_2_O) and incubated for 30 min at room temperature. Ehrlich's Reagent (100 uL) (2.5 g p-dimethylaminobenzaldehyde added to 9.3 mL n-Propanol and 3.9 mL of 70% Perchloric acid) was added and incubated at 65°C for 30 min. The absorbance was then measured at 560 nm and a standard curve generated using commercially available hydroxyproline stock (Sigma-Aldrich, St. Louis, MO).

### 2.10. Statistical Analysis

The values reported are means ± standard error of the mean (SEM). Data were analyzed with Student's *t*-test using GraphPad Prism (GraphPad Software, San Diego CA).

## 3. Results

### 3.1. Nlrp3^−/−^ Mice Are More Susceptible to Alcohol-Induced Liver Injury

We tested the susceptibility of female B6, Nlrp3^−/−^, and Nlrc4^−/−^ mice to alcohol-induced liver injury after chronic administration of Lieber-DeCarli ethanol-containing diet. No differences in daily food intake were found in any of the strains (Table 1). To ensure that the various strains metabolized ethanol in a similar manner, serum ethanol levels were measured 2 hours into the feeding cycle. Nlrc4^−/−^ mice had reduced plasma ethanol concentrations (0.5-fold compared to B6 mice fed ethanol-containing diet), while Nlrp3^−/−^ mice showed similar increased plasma ethanol concentrations compared to B6 mice (Table 1). To confirm that Nlrc4^−/−^ mice metabolized ethanol in a similar manner to the other strains, B6, Nlrp3^−/−^, and Nlrc4^−/−^ mice were given an acute ethanol gavage and blood was taken from the tail vein thirty minutes later (Supplementary Data, Figure 1). Blood alcohol levels were measured and found to be similar between the strains and thus the strains metabolized ethanol in a comparable manner.

**Figure 1:**
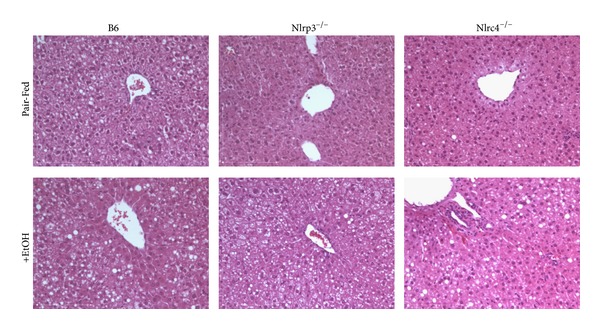
Analysis of steatosis with alcohol feeding. Hematoxylin and Eosin (H&E) staining of livers from C57BL/6J (B6), Nlrp3^−/−^, and Nlrc4^−/−^ mice fed pair-fed control diets (Pair-Fed) or ethanol-containing diets (+EtOH). Figures are representative of 6 mice per group. Original magnification, ×200.

**Figure 2:**
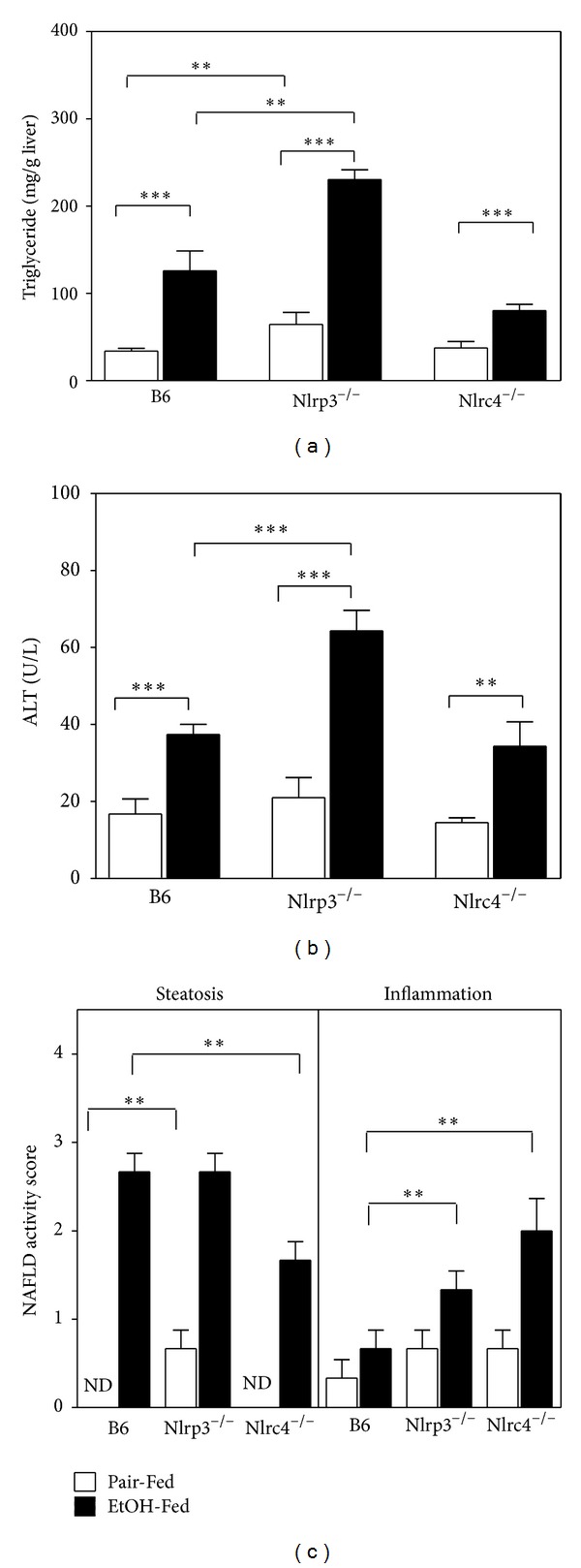
Measurements of liver injury. (a) Hepatic triglycerides were measured biochemically from C57BL/6J (B6), Nlrp3^−/−^, and Nlrc4^−/−^ mice fed the ethanol-containing diet (EtOH-Fed) or pair-fed diet (Pair-Fed). (b) Plasma ALTs were measured with enzymatic assays from mice at the completion of the ethanol feeding trial. (c) NAFLD histological scores for steatosis and inflammation in B6, Nlrp3^−/−^, and Nlrc4^−/−^  mice. Values represent the mean ± SEM with **P < 0.01, ***P < 0.001 by Student's *t*-test for *n* = 4–6 mice per group. ND is abbreviation for NAFLD activity score of 0.

Liver injury was characterized as an increase in hepatic triglyceride and an increase in plasma ALT after ethanol consumption compared to pair-fed controls as well as histological analysis for steatosis and inflammation [36]. At the end of the ethanol-diet study, liver sections were analyzed by H&E staining (Figure 1). All strains had accumulation of lipid droplets with ethanol feeding. The concentration of hepatic triglycerides were measured biochemically and found to be increased in ethanol-fed mice for each strain of mice (Figure 2(a)). Histological analysis for steatosis in liver sections from each strain indicated that Nlrp3^−/−^ mice had greater NAFLD activity scoring for steatosis in the pair-fed animals yet had a similar score as B6 when fed Lieber DeCarli ethanol-containing diet (Figure 2(c)). In contrast, the Nlrc4^−/−^ mice had the lowest NAFLD activity scoring for steatosis after ethanol feeding compared to B6 and Nlrp3^−/−^ mice. To examine inflammation, the pathologist analyzed the stained liver sections and scored them using NAFLD activity scoring [30]. There was mild appearance of inflammation in B6 and Nlrp3^−/−^ mice and Nlrp3^−/−^ mice had greater NAFLD activity scoring for inflammation (Figure 2(c)). The Nlrc4^−/−^ mice, on the other hand, had the highest NAFLD activity scoring for inflammation (Figure 2(c)). To further analyze liver injury plasma ALTs were measured. Liver injury was evident in all mice, as they exhibited increased plasma ALT concentrations. However, Nlrp3^−/−^ mice showed the greatest increase in ALTs (3.0 fold) over its pair-fed control, while Nlrc4^−/−^ and B6 mice had similar increases over their pair-fed controls (2.2-fold and 2.3-fold, resp.) (Figure 2(b)). This data suggests that Nlrp3^−/−^ mice have increased alcohol-induced liver injury with greater plasma ALT, while Nlrc4^−/−^ mice have greater inflammation after alcohol consumption compared to B6 mice.

**Figure 3:**
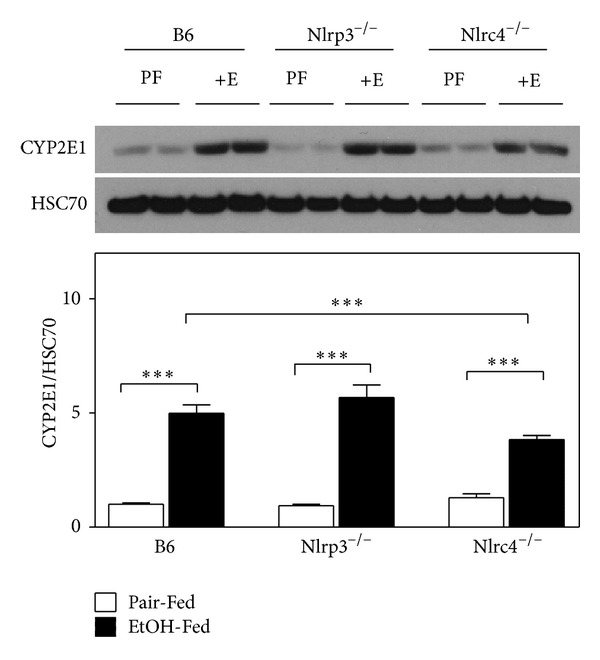
CYP2E1 induction with alcohol consumption. (a) western blot analysis of proteins from livers isolated from C57BL/6J (B6), Nlrp3^−/−^, and Nlrc4^−/−^ mice fed ethanol-containing diet (EtOH-Fed) or pair-fed diet (Pair-fed). Westerns were normalized with heat shock cognate-70 (HSC70) as a loading control. In the graph, densitometric scans of western blots were performed and analyzed. Values represent the mean ± SEM with **P < 0.05, ***P < 0.001 by Student's *t*-test for *n* = 4–6 mice per group.

**Figure 4:**
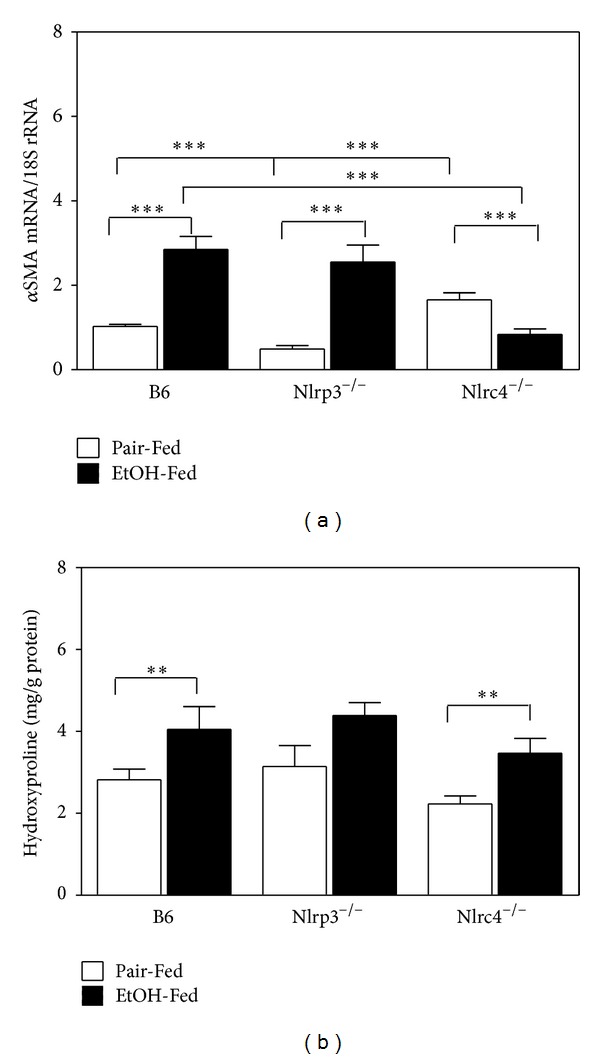
Measurement of *α*SMA and hydroxyproline. Total RNA was isolated from livers of C57BL/6J (B6), Nlrp3^−/−^,  and  Nlrc4^−/−^ mice fed Lieber-DeCarli ethanol-containing diet (+EtOH) or pair-fed controls (Pair-Fed). Expression of (a) *α*SMA mRNA was measured and normalized by 18S rRNA. (b) Hydroxyproline was measured in protein homogenates by ELISA and normalized by protein concentration in the homogenates. The values are the means ± SEM for *n* = 4–6 mice per group. Values represent the mean ± SEM with **P < 0.05, ***P < 0.001 by Student's *t*-test.

Since ethanol consumption induces CYP2E1 expression and activity [37], we measured the induction of CYP2E1 by western blot analysis. All strains had increased expression of CYP2E1 with ethanol feeding (6.2-fold, 7.5-fold, and 3.7-fold over pair-fed controls for B6 and Nlrp3^−/−^ mice and Nlrc4^−/−^ mice, resp.) (Figure 3). The oxidative stress generated by increased CYP2E1 promotes alcohol liver disease and liver fibrosis [38]. In mice, it is difficult to induce frank liver fibrosis with 4 only weeks of alcohol feeding. To determine if deletion of Nlrp3 or Nlrc4 influences known components in development of liver fibrosis, we measured *α*-SMA mRNA and hydroxyproline expression. The *α*-SMA mRNA was induced with alcohol feeding in B6 and Nlrp3^−/−^ mice. However, in Nlrc4^−/−^ mice the basal expression of *α*-SMA mRNA was greater and ethanol feeding resulted in repression of *α*-SMA mRNA (Figure 4). Liver hydroxyproline content was measured using a hydroxyproline assay. In B6 and Nlrc4^−/−^ mice there was an increase in hydroxyproline with ethanol feeding, while the Nlrp3^−/−^ mice had a blunted induction of hydroxyproline (Figure 4(b)). The hydroxyproline values measured in Figure 4 were very low compared to previous studies of mice that had frank fibrosis (~18 mg/g protein for hydroxyproline after treatment with ethanol and carbon tetrachloride) [39].

**Figure 5:**
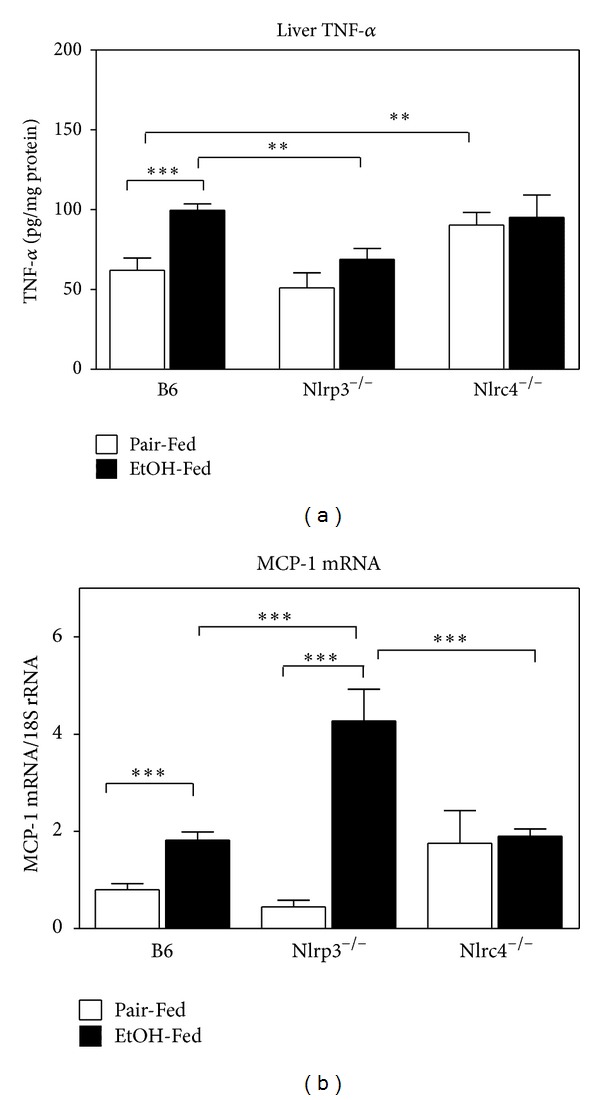
Expression of hepatic TNF-*α*. Protein was isolated from livers of C57BL/6J (B6), Nlrp3^−/−^, and  Nlrc4^−/−^ mice fed Lieber-DeCarli ethanol-containing diet (EtOH-Fed) or pair-fed controls (Pair-Fed). Expression of hepatic TNF-*α* was measured. The values are the means ± SEM and normalized with protein concentration in the homogenate for *n* = 4–6 mice per group. Values represent the mean ± SEM with **P < 0.05, ***P < 0.001 by Student's *t*-test.

It has been proposed that activation of the resident liver macrophages, Kupffer cells, has a pivotal role in the inflammation associated with alcohol liver disease (ALD) by secreting TNF-*α* as well as other cytokines [7, 40]. In addition the chemokine, monocyte chemoattractant protein-1 (MCP-1) also contributes to alcohol-induced fatty liver likely via downregulation of PPAR-*α* and its target fatty acid metabolism genes [41]. Because TNF-*α* and MCP-1 influence the development of ALD, we measured hepatic TNF-*α* and MCP-1 in B6, Nlrp3^−/−^, and Nlrc4^−/−^ mice (Figure 5). B6 mice had increased TNF-*α* with ethanol consumption, but both Nlrp3^−/−^ and Nlrc4^−/−^ did not have an increase of TNF-*α* in response to alcohol. The Nlrp3^−/−^ mice had blunted levels similar to B6 pair-fed mice, while Nlrc4^−/−^ mice had higher levels similar to B6 ethanol-fed mice. For MCP-1 expression, B6 mice had induced levels of MCP-1 with ethanol feeding and Nlrp3^−/−^ mice had a dramatic increase of the chemokine with ethanol feeding (Figure 5(b)). However, Nlrc4^−/−^ had levels similar to B6 ethanol-fed mice of MCP-1 for both pair-fed and ethanol-fed mice.

**Figure 6:**
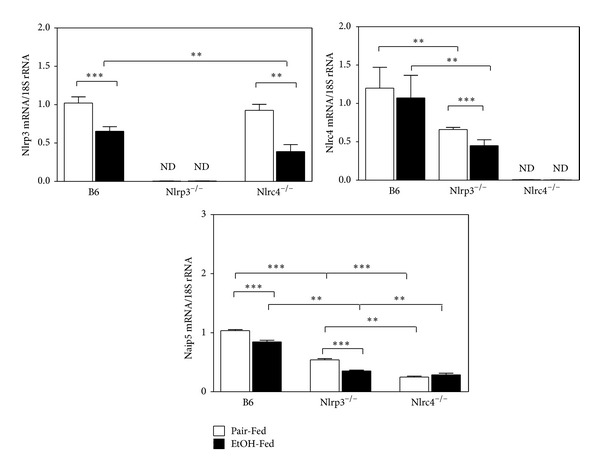
Hepatic expression of Nlrp3, Nlrc4, and Naip5 mRNA. RNA was isolated from C57BL/6J (B6), Nlrp3^−/−^, and Nlrc4^−/−^ mice fed Lieber-DeCarli ethanol-containing diet (EtOH-Fed) or pair-fed controls (Pair-Fed). Expression of Nlrp3, Nlrc4, and Naip5 was measured. The values are the means ± SEM and normalized with 18S rRNA for *n* = 4–6 mice per group. Values represent the mean ± SEM with **P < 0.05, ***P < 0.001 by Student's *t*-test.

IL-1*β* is a potent proinflammatory cytokine that is elevated in patients with ALD [24, 42]. To determine if there was compensation for the deleted NLR molecules, we measured Nlrp3, Nlrc4, and Naip5 mRNA in B6 and knockout mice. In the Nlrp3^−/−^ and Nlrc4^−/−^ mice, the other NLR member of the inflammasome mRNA was reduced (Figure 6). Naip5 is the sensor component of the Nlrc4 inflammasome that specifically recognizes and binds flagellin from pathogenic bacteria such as *Legionella* or *Salmonella *[43]. Naip5 mRNA was found to be reduced in both Nlrp3^−/−^ and Nlrc4^−/−^ mice. This suggests that Nlrp3 expression is not compensating for the loss of Nlrc4 gene expression in Nlrc4^−/−^ mice and Nlrc4 expression is not compensating for loss of Nlrp3 gene expression in Nlrp3^−/−^ mice.

**Figure 7:**
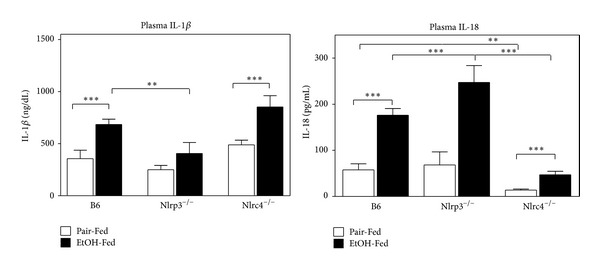
Altered IL-1*β* and IL-18 expression in the liver in Nlrp3^−/−^ and Nlrc4^−/−^ mice. Protein was isolated from livers of C57BL/6J (B6), Nlrp3^−/−^, and Nlrc4^−/−^ mice fed Lieber-DeCarli ethanol-containing diet (EtOH-Fed) or pair-fed controls (Pair-Fed). Levels of IL-1*β* and IL-18 were measured using specific ELISA. Values represent the mean ± SEM with **P < 0.05, ***P < 0.001 by Student's *t*-test for *n* = 4–6 mice per group.

To determine the consequence of deleting Nlrp3 or Nlrc4 genes in the production of proinflammatory cytokines IL-1*β* and IL-18, we measured the active form of these cytokines by ELISA (Figure 7). IL-1*β* was induced in B6 mice fed ethanol, but it was greatly reduced in Nlrp3^−/−^ mice. IL-18 was induced in B6 mice fed ethanol, and it was greatly increased in Nlrp3^−/−^ mice, while IL-18 was reduced in Nlrc4^−/−^ mice. This suggests that Nlrp3 may play a more important role in the activation IL-1*β*, while Nlrc4 may play a more important role in the activation of IL-18.

**Figure 8:**
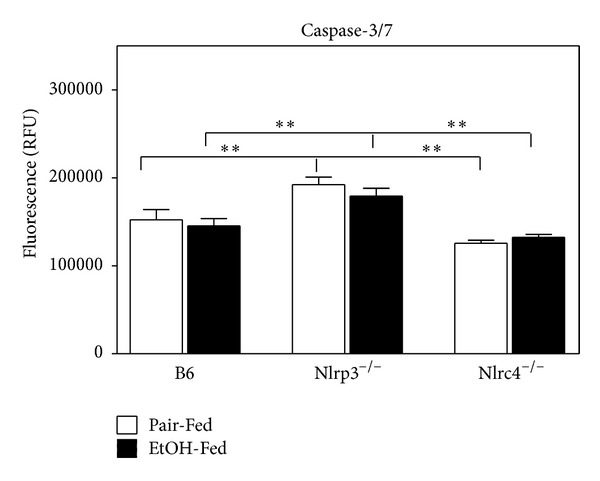
Increased caspase-3/7 in the liver of Nlrp3^−/−^ mice. Protein was isolated from C57BL/6J (B6), Nlrp3^−/−^, and Nlrc4^−/−^ mice fed Lieber-DeCarli ethanol-containing diet (EtOH-Fed) or pair-fed controls (Pair-Fed). Expression of hepatic caspase-3/7 was measured as relative fluorescence. The values are the means ± SEM for *n* = 4–6 mice per group. Values represent the mean ± SEM with **P < 0.05, ***P < 0.001 by Student's *t*-test for *n* = 4–6 mice per group.

**Figure 9:**
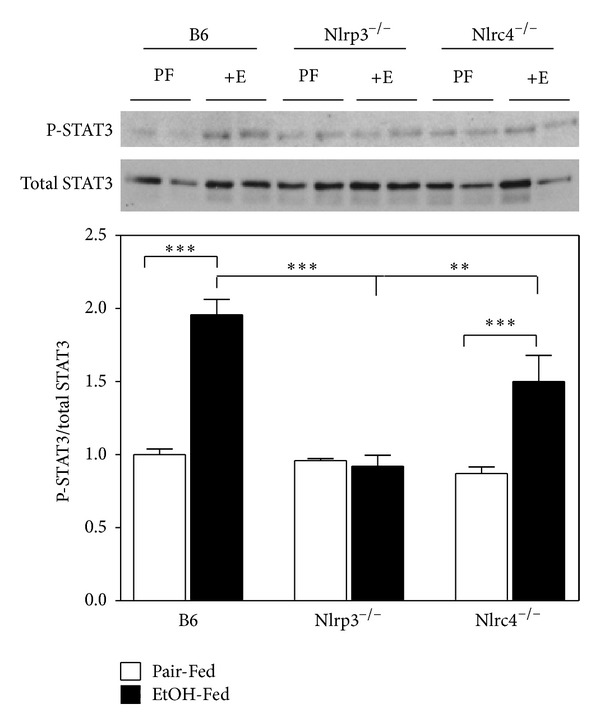
Decreased phosphorylation of STAT3 in the liver in Nlrp3^−/−^ and Nlrc4^−/−^ mice. Protein was isolated from livers of C57BL/6J (B6), Nlrp3^−/−^, and Nlrc4^−/−^ mice fed Lieber-DeCarli ethanol-containing diet (EtOH-Fed) or pair-fed controls (Pair-Fed). Expression of hepatic total STAT3 and phosphorylated STAT3 was measured by western blot analysis. In the graph, densitometric scans of western blots were performed and analyzed. The values are the means ± SEM. The phosphorylated STAT3 was normalized by total STAT3. Values represent the mean ± SEM with **P < 0.05, ***P < 0.001 by Student's *t*-test for *n* = 4–6 mice per group.

Caspase-3 is activated in the apoptotic cell both by extrinsic (death ligand) and intrinsic (mitochondrial) pathways [44]. Since Nlrp3^−/−^ mice have elevated serum ALT, we measured caspase-3/7 activity (Figure 8). Nlrp3^−/−^ mice had a 2-fold increase of caspase-3/7 activity suggesting increased apoptosis in both pair-fed and after ethanol feeding. Cell death can also be a result of decreased liver regeneration. For liver regeneration to occur, Kupffer cells release TNF-*α* and IL-6 which then activate STAT3 phosphorylation and initiate hepatocyte regeneration [45]. We measured STAT3 phosphorylation in B6, Nlrp3^−/−^, and Nlrc4^−/−^ pair-fed and ethanol-fed mice (Figure 9). The Nlrp3^−/−^ mice did not have increased phosphorylation of STAT3 with ethanol feeding and Nlrc4^−/−^ had reduced phosphorylation of STAT3 compared to B6 mice, suggesting that the liver regeneration pathway may also be impaired in these knockout mice.

## 4. Discussion

Previous work has studied the role of Nlrp3 and another member of the NLR inflammasome family, Nlrp6, in the context of nonalcoholic fatty liver disease (NAFLD) [46]. Using a variety of mice deficient for Nlrp3 gene (Nlrp3^−/−^), Nlrp6^−/−^ gene (Nlrp6^−/−^), ACC (ASC^−/−^), IL-18 (IL-18^−/−^), and caspase-1 (caspase-1^−/−^) the role of the inflammasome was investigated. The mice were fed methionine choline deficient diet (MCDD) to induce NASH. The investigators found that Nlrp6 and Nlrp3 inflammasomes and its downstream target IL-18 modulate the development of MCDD-induced liver injury. Because inflammasomes can also act as sensors and regulators of colonic microbiota [47], Mejia et al. analyzed the effects of gut microbiota in the development of NASH using these genetically modified strains. Antibiotic treatment with ciprofloxacin and metronidazole abolished the gut microbiota associated activity with development of NASH in ASC^−/−^ mice. In addition cohousing ASC^−/−^ or IL-18^−/−^ mice with wild-type mice for 4 weeks before feeding MCDD diet to transfer microbiota from one strain to the other resulted in more severe liver injury in the wild-type mice compared to singly housed wild-type mice [46]. However not all NLR deficient mice developed liver injury in the same manner. Henao-Mejia et al. [46] also cohoused Nlrc4^−/−^ and Nlrp12^−/−^ mice with wild-type mice, but these strains did not alter the severity of liver disease with MCDD. This suggests a potential role for the Nlrp3 and Nlrp6 inflammasomes altering gut microbiotica that may in turn alter the development of nonalcoholic induced liver injury.

We have previously reported that the genetic contribution for the development of alcoholic steatohepatitis (ASH) and nonalcoholic steatohepatitis (NASH) is unique [48] and multifactorial. In ALD, LPS derived from gut microflora has been extensively studied as a key inducer of inflammation in alcohol-related conditions. Alcohol stimulates LPS translocation across the gut via a number of mechanisms, and alcoholics with liver diseases are known to have significantly elevated circulating LPS [49]. In mice fed the Lieber DeCarli ethanol-containing diet, alcohol-induced liver injury is associated with increased plasma endotoxin (LPS) and hepatic lipid peroxidation. Treatment with an endotoxin neutralizing protein significantly suppressed alcohol-induced elevation of plasma endotoxin, hepatic lipid peroxidation, and inhibited TNF-alpha production  [50]. These studies suggest the importance of the gut microbiotica and gut permeability in producing LPS in the plasma during ethanol consumption.

In the current study we analyzed the role of two NLR inflammasomes, Nlrp3 and Nlrc4, in the development of ALD. Recent studies have investigated the critical importance of IL-1 signaling in ALD [51] using caspase-1^−/−^, ASC^−/−^, or IL-1 receptor knockout mice (IL-1R^−/−^). Loss of downstream signaling resulted in attenuation of alcohol-induced liver inflammation, steatosis, and damage [51]. The role of IL-18 in development of ALD has been studied in the context of a combined insult of ethanol and burn injury. In mice, the combined insult resulted in the suppression of immune responses with decreased host resistance and enhanced susceptibility to infection [52, 53]. However, the role of Nlrp3 and Nlrc4 inflammasomes in the development of ALD has not been fully elucidated.

Since previous studies showed that caspase-1 mediated activation of IL-1*β* was required for ALD, we hypothesized that deletion of either Nlrp3 or Nlrc4 genes would prevent ALD. Yet Nlrc4^−/−^ mice had similar alcohol-induced injury compared to B6 mice, while Nlrp3^−/−^ had more severe alcohol-induced liver injury compared to B6 mice (Figure 2(b)). In Nlrp3^−/−^ mice, the loss of the Nlrp3 inflammasome reduced the amount of active IL-1*β* but dramatically increased the amount of active IL-18. This result is different from previously published studies that have shown that IL-18 was not induced in B6 mice fed ethanol [51]. Our results may be different because the B6 mice used in our study had been bred within an animal colony at Case Western Reserve University for over eight generations, resulting in slightly different inbred strains from Jackson labs. The other possibility is that Petrasek et al. initiated their ethanol feeding study in 6–8-week-old female mice, while we began our feeding study in 10–12-week-old adult female mice. In addition as Henao-Mejia et al. [46] have shown, Nlrp3 inflammasome but not the Nlrc4 inflammasome impacts microbiota in the gut which in turn modulated development of NASH with MCDD. This could also be a mechanism by which Nlrp3^−/−^ mice have increased liver injury with alcohol consumption, while Nlrc4^−/−^ mice have similar injury to B6 mice in our study. Nlrp3^−/−^ mice may have increased leakage of LPS from the gut possibly due to altered microbiota that would impact the degree of liver injury. Further studies are needed to fully understand the role of Nlrp3 inflammasome and its impact on microbiota in the gut with alcohol feeding.

Could the increase of IL-18 contribute to the increased ALD in Nlrp3^−/−^ mice? Finotto et al. analyzed IL-18 transgenic mice that expressed IL-18 under the control of CD2 promoter (express in T cells and B cells) [54]. The transgenic mice had increased hepatocyte apoptosis by spontaneous activation of the Fas associated death pathway [54]. Binding of IL-18 to the high affinity IL-18R leads to nuclear factor *κβ* (NF*κβ*) activation through myeloid differentiation primary response 88 (MyD88) and TNF-*α* and subsequent phosphorylation of I*κβ* via I*κβ* kinases (IKK-1 and IKK-2). IL-18 also triggers NK cell activity and expression of FasL by natural killer cells (NK) [55, 56]. In our study, we suggest that Nlrp3 plays a key role in production of IL-1*β* and loss of the Nlrp3 inflammasome increases hepatocyte apoptosis possibly through FasL mediated mechanism.

The decrease of IL-1*β* may also impact cell survival. Previous studies have shown that active IL-1*β* but not IL-18 is induced after ethanol feeding in mice [51]. Could the lack of IL-1*β* contribute to increase ALD in Nlrp3^−/−^ mice? IL-1*β* is thought to mediate its inflammatory actions by inducing the expression of proinflammatory genes (such as IL-6), recruiting immune cells to the site of injury (liver), and modulating infiltrating cellular immune-effector actions [57]. The proinflammatory cytokine, IL-1*β*, exerts a prominent effect on the expression of proinflammatory genes primarily by activation of intracellular signaling pathways involving NF-*κβ* and p38 mitogen-activated protein kinase (MAPK) [58, 59]. The transcription factor NF-*κβ* is inactive when associated with the inhibitory protein I*κβ*. Upon cytokine activation, I*κβ* is degraded and NF-*κβ* translocates to the nucleus [60]. Both NF-*κ*B and p38 MAPK are involved in the regulation of the expression of genes encoding E-selectin, vascular cell adhesion molecule-1 (VCAM-1), intercellular adhesion molecule 1 (ICAM-1), IL-6, IL- 8, and cyclooxygenase (COX)-2 [61–63]. When IL-6 binds to its receptor, gp130 is dimerized and associates with Janus kinases (JAKs) and phosphorylation of JAKs and gp130 occurs. This receptor-kinase complex then recruits and phosphorylates cytoplasmic STAT3. Once phosphorylated, STAT3 forms a dimer and translocates into the nucleus initiating transcription of many genes that play significant roles in inducing acute phase responses, promoting hepatocyte survival and liver regeneration [64]. In the present study we found blunted phosphorylation of STAT3 in response to ethanol in Nlrp3^−/−^ mice (Figure 9). In addition the amount of caspase-3/7 was dramatically increased in Nlrp3^−/−^ mice due to the loss of Nlrp3 (Figure 8). Therefore, we conclude that the Nlrp3 inflammasome contributes to the activation of JAK/STAT3 pathway and may promote liver regeneration. However further studies are needed to fully understand the mechanism for increased ethanol-induced liver injury in Nlrp3^−/−^ mice.

Since both Nlrp3 and Nlrc4 inflammasomes activate caspase-1 and produce IL-1*β* and IL-18, can these pathways have nonredundant roles? As stated above, each has their own activators and Nlrc4 has a narrower spectrum of activators, primarily flagellin. In a study that infected bone marrow derived macrophages with* Burkholderia pseudomallei* (Gram-negative bacteria), differences in activation of inflammasomes and the amount of active IL-1*β* and IL-18 produced were found [65]. Using B6, Nlrp3^−/−^,  Casp1^−/−^, Nlrc4^−/−^, and ASC^−/−^ mice, Ceballos-Olvera et al. found that Nlrc4 contributes to IL-1*β* production in the early phase of infection. This is important for early induction of pyroptosis, which would then restrict bacterial growth. Nlrp3 does not regulate pyroptosis and primarily controls IL-1*β* secretion. Most importantly they found that IL-1*β* and IL-18 were present at high levels in lungs of Nlrc4^−/−^ mice that were infected with *B. pseudomallei *intranasally. In contrast, Nlrp3^−/−^ and ASC^−/−^ mice had little to no IL-1*β* produced after infection [65]. What determines this specificity? In this same study, the authors suggest the Nlrc4 can form two distinct Nlrc4 inflammasomes, one Nlrc4 inflammasome that contains ASC and regulates IL-1*β* production and the other lacking ASC which would activate caspase-1 and initiate pyroptosis [65, 66]. Finally members of NLR family, Naip family, have been shown to determine the specificity of Nlrc4 for its activators [67]. For example, activation of Nlrc4 inflammasome by bacterial PrgJ from *Salmonella* Typhimurium requires Naip2, while activation of Nlrc4 by flagellin from L. *pneumophila *requires Naip5 [67]. In the present study, we found that Nlrp3^−/−^ mice had reduced formation of active IL-1*β* with increased formation of active IL-18. In Nlrc4^−/−^ mice, the reverse was found. Nlrc4^−/−^ mice had more active IL-18 but less active IL-1*β*. This data supports the hypothesis that each inflammasome may preferentially produce either active IL-1*β* or IL-18 adding to the complexity of regulation in the development of ALD.

## 5. Conclusions

In summary, we present evidence that Nlrp3 inflammasome is protective during alcohol-induced liver injury. The data presented in this study analyzed whole liver homogenates, but the liver is composed of several cell types (hepatocytes, Kupffer cells, NK cells, endothelial cells, and hepatic stellate cells). Because previous studies have shown the importance of Nlrp3 and ASC in hepatic stellate cells for the development of liver fibrosis [68], future studies will determine the role of the Nlrp3 inflammasome in the specific cell types for the development of ALD.

## Supplementary Material

Supplementary figure contains the blood alcohol levels of B6, Nlrp3^−/−^ and Nlrc4^−/−^ mice thirty minutes after alcohol gavage.
Supplementary table contains the specific primer sequences used for qRT-PCR.

## Figures and Tables

**Table 1:** 

	C57BI/6J		NIrp3−/−		NIrc4−/−	
	PF	+E	PF	+E	PF	+E
Food intake (mL/mouse/day)	12.51 ± 0.3^a^	12.27 ± 0.2^a^	12.6 ± 0.2^a^	12.8 ± 0.5^a^	12.56 ± 0.27^a^	12.27 ± 02^a^
Blood alcohol (mM)	0.42 ± 0.08^a^	62.49 ± 15.74^b^	0.08 ± 0.03^c^	54.51 ± 2.72^b^	0.29 ± 0.05^d^	30.58 ± 4.83^e^

Values are the mean ± SEM for *n* = 4–6 female mice per group. The means in a row with superscripts without a common letter differ from each other, *P* < 0.05 as determined with ANOVA and Bonferroni's correction for multiple testing. (PF: pair-fed, +E: ethanol-fed diet).
