# Supplementary material for: Alcohol-Induced Liver Injury Is Modulated by Nlrp3 and Nlrc4 Inflammasomes in Mice
Source: Mediators Inflamm. 2013 Dec 16;2013:751374. doi: 10.1155/2013/751374 (PMC3876912; doi:10.1155/2013/751374)
Supplement: Supplementary file 1 — Supplementary figure contains the blood alcohol levels of B6, Nlrp3−/− and Nlrc4−/− mice thirty minutes after alcohol gavage. Supplementary table contains the specific primer sequences used for qRT-PCR. [file 751374.f1.pdf]

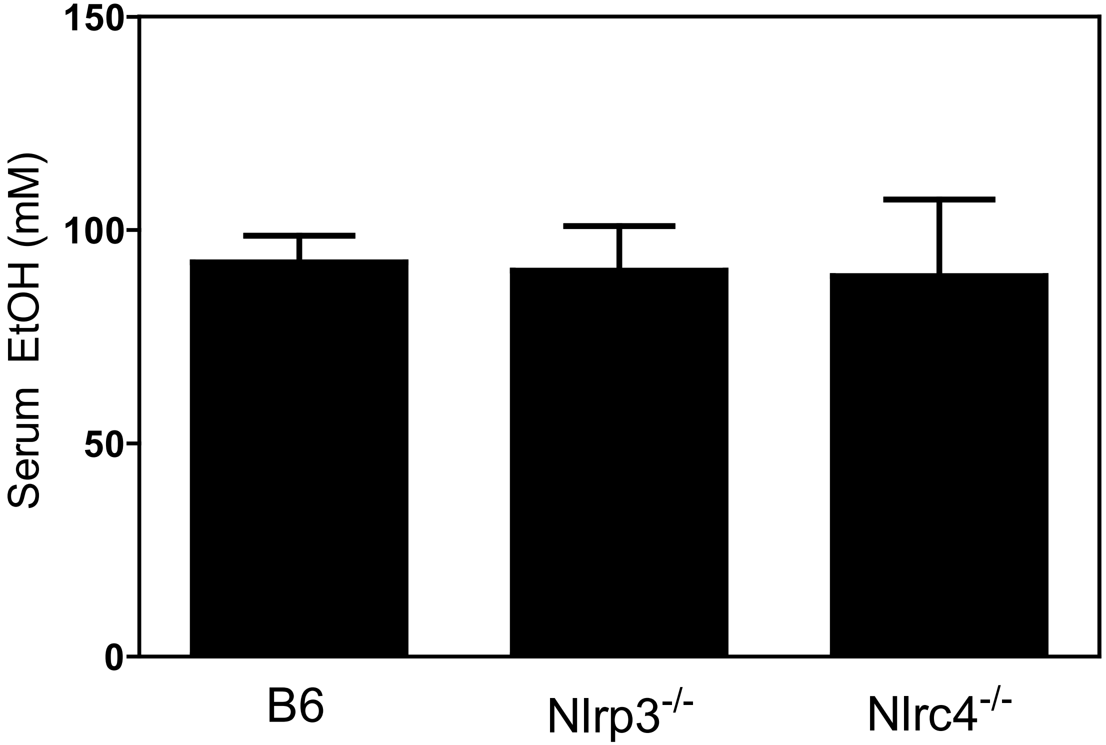
150
100
50
0
Serum EtOH (mM)
B6
Nlrp3-/-
Nlrc4-/-

Gene	Forward Primer	Reverse Primer
α-SMA	5’ctgacagaggcaccactgaa	5’agaggcatagagggacagca
Nlrc4	5’aattcagatgggcagacagg	5’tcacctgaagctccacctct
Nlrp3	5’attacccgcccgagaaagg	5’catgagtgtggctagatccaag
Naip5	5’tgcagctgactctctctcca	5’gatgcacaaccacatcaagg
MCP-1	5’aggtccctgtcatgcttctg	5’cgttaactgcatctggctga
18S rRNA	5’ccatccaatcggtagtagcg	5’gtaacccgttgaaccccatt
